# Higher sensitivity of *pad2-1* and *vtc2-1* mutants to cadmium is related to lower subcellular glutathione rather than ascorbate contents

**DOI:** 10.1007/s00709-013-0576-x

**Published:** 2013-11-27

**Authors:** Barbara Eva Koffler, Lisa Polanschütz, Bernd Zechmann

**Affiliations:** Institute of Plant Sciences, University of Graz, Schubertstrasse 51, 8010 Graz, Austria

**Keywords:** Arabidopsis, Ascorbate, Cadmium, Glutathione, Transmission electron microscopy

## Abstract

Cadmium (Cd) interferes with ascorbate and glutathione metabolism as it induces the production of reactive oxygen species (ROS), binds to glutathione due to its high affinity to thiol groups, and induces the production of phytochelatins (PCs) which use glutathione as a precursor. In this study, changes in the compartment specific distribution of ascorbate and glutathione were monitored over a time period of 14 days in Cd-treated (50 and 100 μM) Arabidopsis Col-0 plants, and two mutant lines deficient in glutathione (*pad2-1*) and ascorbate (*vtc2-1*). Both mutants showed higher sensitivity to Cd than Col-0 plants. Strongly reduced compartment specific glutathione, rather than decreased ascorbate contents, could be correlated with the development of symptoms in these mutants suggesting that higher sensitivity to Cd is related to low glutathione contents rather than low ascorbate contents. On the subcellular level it became obvious that long-term treatment of wildtype plants with Cd induced the depletion of glutathione and ascorbate contents in all cell compartments except chloroplasts indicating an important protective role for antioxidants in chloroplasts against Cd. Additionally, we could observe an immediate decrease of glutathione and ascorbate in all cell compartments 12 h after Cd treatment indicating that glutathione and ascorbate are either withdrawn from or not redistributed into other organelles after their production in chloroplasts, cytosol (production centers for glutathione) and mitochondria (production center for ascorbate). The obtained data is discussed in respect to recently proposed stress models involving antioxidants in the protection of plants against environmental stress conditions.

**Electronic supplementary material:**

The online version of this article (doi:10.1007/s00709-013-0576-x) contains supplementary material, which is available to authorized users.

## Introduction

Contaminations of soils with heavy metals such as cadmium (Cd) are a severe problem for plant growth and development as they accumulate and negatively interfere with many physiological processes in plants. Environmental pollution by metals such as Cd became extensive in the late 19th and early 20th century as mining and industrial activities increased (Benavides et al. 2005; Gallego et al. 2012). Anthropogenic sources like zinc smelting, burning of fuel, waste incinerators, urban traffic, cement factories, and Cd as a by-product of phosphate fertilizers have led to Cd pollution in the environment (Sanità di Toppi and Gabbrielli 1999; DalCorso et al. 2008; Jozefczak et al. 2012). Although Cd is not an essential element, it is readily absorbed by the roots and transported through xylem and phloem to different parts of the plants (Mendoza-cózatl et al. 2008; DalCorso et al. 2008). Visible symptoms of Cd toxicity in plants are leaf roll, chlorosis, browning of root tips and reduced growth (Baryla et al. 2001; Schützendübel and Polle 2002; Chen et al. 2003; Gratão et al. 2005; Maughan et al. 2010). Inside the plant Cd leads to an alteration of the chloroplast ultrastructure, disturbs the synthesis of chlorophyll and carotenoids, inhibits the enzyme activity of the Calvin cycle and leads to CO_2_ deficiency due to stomatal closure, which cause the inhibition of photosynthesis (Ding et al. 2006; Ekmekci et al. 2008; He et al. 2008; Gratão et al. 2009; Bouzon et al. 2012). Additionally, Cd causes lipid peroxidation, disrupts cell transport processes, reduces the uptake of essential mineral nutrients, reduces the activity of various enzymes and is responsible for chromosomal aberrations (Clemens et al. 2001; Schützendübel and Polle 2002; Ding et al. 2006; Sanità di Toppi et al. 2008; Gratão et al. 2012). Indirectly, high concentrations of Cd lead to oxidative stress which activates the accumulation of anti-oxidative defense enzymes and antioxidants like ascorbate and glutathione (Sanità di Toppi and Gabbrielli 1999; Hegedüs et al. 2001; Gratão et al. 2005; Paradiso et al. 2008; Ekmekci et al. 2008; Xu et al. 2008; DalCorso et al. 2008; Gill and Tuteja 2010; Yadav 2010; Monteiro et al. 2011; Gallego et al. 2012; Shan et al. 2012; Zelinova et al. 2013) which detoxify reactive oxygen species (ROS) either through reactions catalyzed by peroxidases or through the ascorbate glutathione cycle (Foyer and Noctor 2005; Iannone et al. 2010; Foyer and Noctor 2011; Noctor et al. 2012). In addition, Cd has a high affinity to thiol groups and forms complexes with reduced glutathione which are then transported into vacuoles. Glutathione is also able to protect proteins by reversible binding to their SH groups which protects them from oxidation by ROS and inhibits the binding of Cd (Rauser 2001; Maksymiec and Krupa 2006; Semane et al. 2007; DalCorso et al. 2010; Jozefczak et al. 2012). On top glutathione serves as a direct precursor of phytochelatins (PCs), which together with metallothioneins detoxify Cd by complexation, transportation, and deposition in the vacuole (Akhter et al. 2012).

Considering the importance of ascorbate and glutathione in the protection against Cd changes in the contents of these antioxidants are well documented in Cd-treated plants. After treatment with 10 μM Cd sulfate for 24 h, *Arabidopsis thaliana* plants showed reduced ascorbate contents in roots (Smeets et al. 2009) and leaves (Keunen et al. 2013). While experiments with *Brassica juncea* showed increased levels of ascorbate in roots after Cd treatment (Mohamed et al. 2012), decreased ascorbate levels were found after long-term Cd exposure of *Ceratophyllum demersum* (Aravind and Prasad 2005), *Brassica campestiris* (Anjum et al. 2008) and *Bechmeria nivea* (L.) Gaud (Liu et al. 2007). Glutathione levels in Cd-treated plants were found to be decreased, e.g., in soybean leaves (Noriega et al. 2012), in cotyledons and embryonic axes of pea seed (Smiri et al. 2011), in Cd sensitive rice seedlings (Chao et al. 2011), in *B. campestiris* leaves (Anjum et al. 2008), and within glandular trichomes of *Cucurbita pepo* (Kolb et al. 2010). Other studies revealed either unchanged glutathione contents for example within Cd tolerant rice seedlings (Chao et al. 2011), or increased glutathione levels like in *B. juncea* shoots and roots (Mohamed et al. 2012), in leaves of *Bechmeria nivea* (Liu et al. 2007) and in Arabidopsis plants (Wójcik and Tukiendorf 2011). Even though these investigations paint a clear picture about the importance and functions of ascorbate and glutathione in the protection against Cd the results of these studies differ in time points, Cd concentration, plant species as well as plant organs and tissues. In particular, the situation during Cd exposure over a period of 14 days in Arabidopsis remains unclear. As stress responses in plants strongly depend on plant species, the state of acclimation and adaptation, severity and duration of stress (Tausz et al. 2004; Gratão et al. 2008; Kranner et al. 2010), it is important to monitor different severities of stresses at different time points in one plant species in order to get a deeper insight into the stress response of a plant. Additionally, Cd-induced stress responses in plants have mainly been investigated by using biochemical methods in order to investigate ascorbate and glutathione contents in whole leaves or organs (Liu et al. 2007; Anjum et al. 2008; Chao et al. 2011; Smiri et al. 2011; Wójcik and Tukiendorf 2011; Noriega et al. 2012; Mohamed et al. 2012). Thus, results about the situation concerning glutathione or ascorbate contents are rarely available for individual cell compartments (Kolb et al. 2010). Since Cd enters the cytosol first and affects this cell compartment stronger than others compartment specific data of ascorbate and glutathione contents could give valuable information about the subcellular importance of ascorbate and glutathione in the protection against Cd.

Thus, the aim of this study was to investigate the compartment-specific distribution of ascorbate and glutathione in Arabidopsis plants during Cd exposure. Different concentrations and time points of Cd treatment were investigated over a period of 14 days in order to clarify the dynamic compartment specific protection of these key antioxidants against Cd exposure. Additionally, we compared the situation in the ascorbate and glutathione deficient mutant *vtc2-1* (60 % less ascorbate than Col-0; Zechmann et al. 2011c) and *pad2-1* (80 % less glutathione than Col-0; (Parisy et al. 2006; Zechmann et al. 2008), respectively, in order to clarify how low ascorbate and glutathione contents contribute towards Cd sensitivity.

## Materials and methods

### Plant material and growth conditions

After stratification for 4 days at 4 °C, seeds of *A. thaliana* accession Col-0 and two Arabidopsis mutants (*pad2-1*, *vtc2-1*) were grown in a mixture of vermiculite and sand (2:1) in growth chambers with a 10/14 h day/night photoperiod. Temperatures were 22 °C during daytime and 18 °C at night. The relative humidity was 60 % and the relative soil water content was kept at 100 %. The light intensity was 150 μM m^−2^ s^−1^.

For Cd treatment, 6- to 8-week-old plants were removed from the pots and were gently washed with water to eliminate vermiculite and sand from the roots. The plants were transferred into plastic dishes (1,500 ml) covered with a nylon mesh that held up the stems and leaves whereas the roots immersed into the solution. Oxygen was supplied to the solution through an aquarium pump. For treatment the dishes were filled either with nutrient solution as control or nutrient solution mixed with Cd sulfate (50 and 100 μM). Cd treatment was performed for different time points (12 h, 24 h, 48 h, 96 h, 7 days and 14 days) with plants of different age so that by the time of harvesting, all plants were 8 weeks old. The solution contained 5 mM KNO_3_, 1 mM KH_2_PO_4_, 2 mM Mg(NO_3_)_2_⋅6H_2_O, 2.5 mM CaSO_4_⋅2H_2_O, 1 mM MgSO_4_⋅7H_2_O, 70 μM EDTA-FeNa, 4 mM Ca(NO_3_)_2_⋅4H_2_O, 0.9 μM ZnCl_2_, 30 μM H_3_BO_3_, 0.9 μM CuCl_2_⋅2H_2_O, 0.5 μM MoO_3_, 20 μM MnCl_2_⋅4H_2_O and was as well as the Cd solution changed every 4 days. The pH value of the solution was adjusted to 6.5.

### Sample preparation for transmission electron microscopy

Two hours after the onset of the light period the center of three different leaves (always taken from the fourth rosette) from each sample were cut into 1.5-mm^2^ pieces on a modeling wax plate in a drop of 2.5 % paraformaldehyde/0.5 % glutardialdehyde in 0.06 M Sørensen phosphate buffer (pH 7.2). The samples were then transferred into glass vials filled with the same fixative for 90 min at room temperature (RT) and were then dehydrated with increasing concentrations of acetone (50 %, 70 %, 90 %). Subsequently, specimens were infiltrated with increasing concentrations of LR-White resin (30 %, 60 %, 100 %; London Resin Company Ltd., Berkshire, UK) mixed with acetone (90 %) for at least 3 h at each step at RT. Finally, the samples where embedded in pure, fresh LR-White resin and polymerized at 50 °C for 48 h in small plastic cups under anaerobic conditions. Ultrathin sections of about 80 nm were generated by cutting the samples with a Reichert Ultracut S ultramicrotome (Leica Microsystems, Wetzlar, Germany).

### Immunogold labeling of ascorbate and glutathione

Cytohistochemical investigation were performed as described previously (Zechmann et al. 2011a, b, c). Ultrathin sections were blocked with 2 % bovine serum albumin (BSA; Sigma-Aldrich, St. Louis, MO, USA) in phosphate buffered saline (pH 7.2). Subsequently the samples were treated with the primary antibodies against ascorbate (anti-ascorbate rat polyclonal IgG; Abcam plc, Cambridge, UK) diluted 1:300 in phosphate buffered saline containing 1 % BSA and glutathione (anti-glutathione rabbit polyclonal IgG, Millipore Corp., Billerica, Ma, USA) diluted 1:50 in phosphate buffered saline with 1 % goat serum for 2 h at RT. Then the specimens where rinsed three times with phosphate buffered saline containing 1 % BSA and treated with a 10-nm gold-conjugated secondary antibody (goat anti-rat IgG for ascorbate labeling and goat anti-rabbit IgG [British BioCell International, Cardiff, UK] for glutathione labeling) diluted 1:100 for ascorbate and 1:50 for glutathione in phosphate-buffered saline containing 1 % BSA for 90 min at RT. Next, the samples were washed two times in distilled water and post-stained with uranyl acetate (2 % dissolved in aqua bidest) for 20 s. The samples were analyzed with a Philips CM10 transmission electron microscope where micrographs of randomly photographed immunogold-labeled sections at 8,900-fold magnification were taken. Micrographs of randomly photographed immunogold labeled sections were digitized and gold particles were counted automatically using the software package Cell D with the particle analysis tool (Olympus, Life and Material Science Europa GmbH, Hamburg, Germany) in different visually identified and manually traced cell structures (mitochondria, plastids, nuclei, peroxisomes, the cytosol, vacuoles). Unspecific background labeling was determined on the sections (outside the specimen) and subtracted from the values obtained in the sample. A minimum of 20 (peroxisomes and vacuoles) to 60 (other cell structures) sectioned cell structures of at least 15 different cells were analyzed for gold particle density per sample. For statistical evaluation at least two different samples for each treatment (*n* = 20 for peroxisomes and vacuoles, *n* = 60 for other cell compartments) were analyzed to gain the number of gold particles per μm^2^ per compartment. Differences in gold particle density of Cd-treated and control samples were calculated in percent. Control plants showed similar (not significantly different) subcellular ascorbate and glutathione contents throughout the experiment. Thus, a mean of the determined subcellular ascorbate and glutathione contents was calculated, defined as control and used for calculating significant differences between Cd-treated and control samples. The obtained data were statistically evaluated using Statistica (Stat-Soft Europe, Hamburg, Germany). For statistical analyses, the Mann–Whitney *U* was applied.

Several negative controls were made to support the specificity of the immunogold procedure. Negative controls were treated either with (1) gold conjugated secondary antibody (goat anti-rat IgG for ascorbate and goat anti-rabbit IgG for glutathione) without prior incubation of the section with the primary antibodies, (2) non-specific secondary antibody (goat anti rabbit IgG for ascorbate and goat anti rat IgG for glutathione), (3) preimmune serum instead of the primary antibody and (4) primary antibody against ascorbate and glutathione pre-adsorbed with an excess of reduced and oxidized ascorbate and glutathione, respectively, for 2 h prior to labeling of the sections. For the latter, a solution containing either 10 mM of ascorbic acid, dehydroascorbic acid, reduced or oxidized glutathione was incubated with or without 0.5 % glutardialdehyde for 1 h. When glutardialdehyde was used then its excess was saturated by incubation for 30 min in a solution of 1 % (w/v) BSA. The resulting solutions were both used in independent experiments to saturate the anti-ascorbate and glutathione antibodies for 2 h prior to its use in the immunogold labeling procedure described above. Labeling on sections treated as negative controls showed no or only very little gold particles bound to ascorbate and glutathione which was similar to previous results obtained by using the same methods in different plant species (Zechmann and Müller 2010; Zechmann et al. 2011c). We have shown in previous reports that the antibody against glutathione binds to free reduced and oxidized glutathione and that it does not bind to glutathione conjugated with monochlorobimane through the SH group and glutationylated proteins (Zechmann et al. 2008; Queval et al. 2011). Additionally, it does not bind to PCs as it binds to the amino group and both carbonyl groups of glutathione — the latter are not available for the antibody in PCs (personal communication with Signature Immunologics Inc.). The glutathione and ascorbate status in the glutathione and ascorbate deficient mutants used in this study (*pad2-1* and *vtc2-1*, respectively) has been studied extensively in previous studies and showed a good correlation between data obtained on the subcellular level and data obtained on the whole leaf level with biochemical methods (Parisy et al. 2006; Zechmann et al. 2008; Zechmann and Müller 2008; Zechmann et al. 2011c). The immunogold localization of ascorbate in mutants deficient in ascorbate (*vtc2-1* and *vtc2-2*) revealed a strong decrease of subcellular ascorbate specific labeling between 50 % and 60 % when compared to *A. thaliana* Col-0 plants. This data correlated well with biochemical measurements which revealed a similar decrease of ascorbate contents in whole leaves of these mutants (Zechmann et al. 2011c). The specificity and accuracy of the immunogold labeling method for glutathione was demonstrated on glutathione deficient mutants *pad2-1* and *rml1*, which both showed a strong decrease of compartment-specific glutathione labeling of up to 91 % and 98 %, respectively. This data correlated well with biochemical measurements of glutathione in these mutants revealing a similar decrease in whole leaves of these mutants (Vernoux et al. 2000; Cairns et al. 2006; Parisy et al. 2006).

## Results

### Visual symptoms

**Fig. 1:**
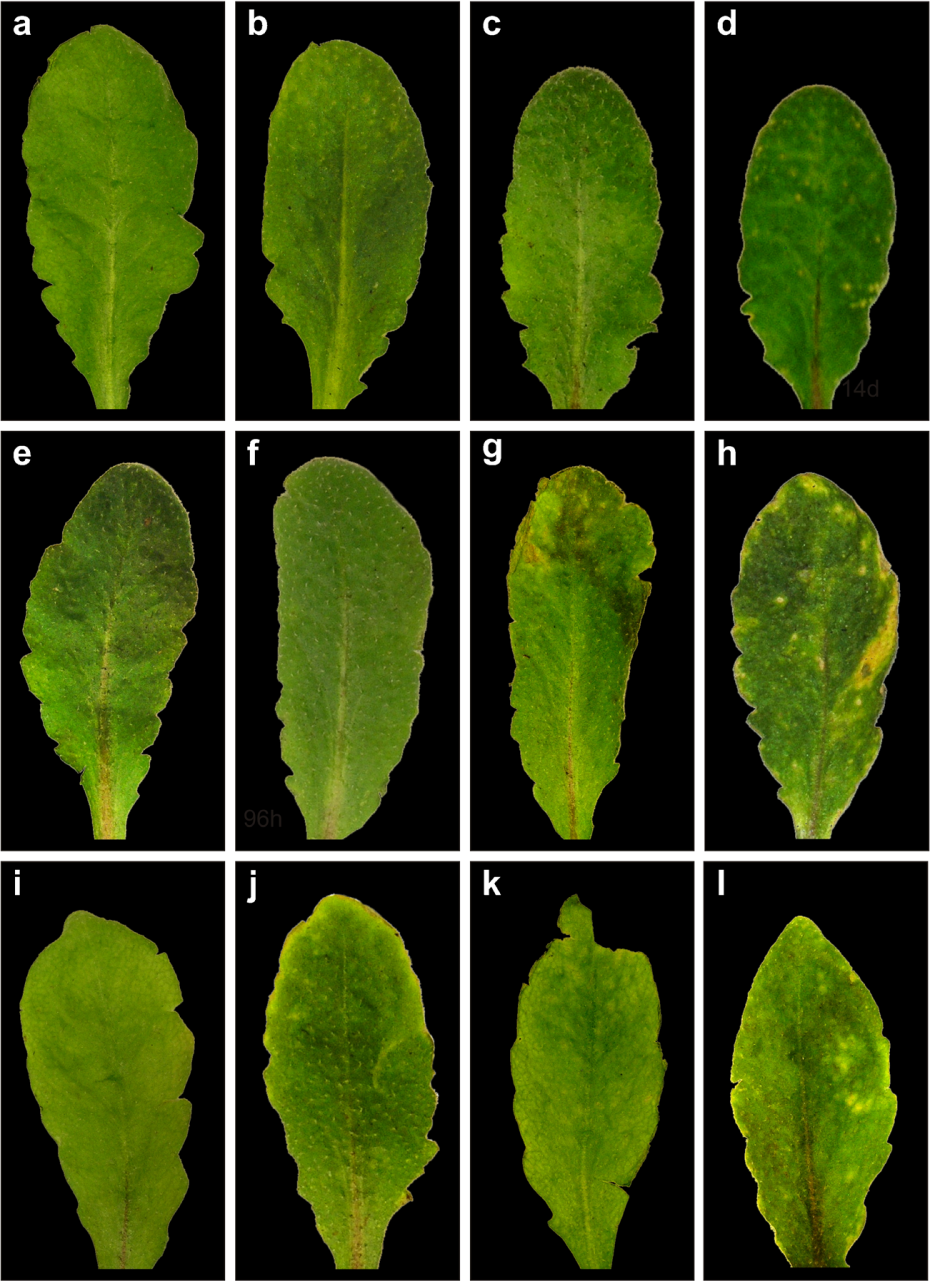
Representative images of leaves from *Arabidopsis thaliana* Col-0 (**a–d**), and the mutants *pad2-1* (**e–h**) and *vtc2-1* (**i–l**) treated with 50 μM of Cd for 0 (**a**, **e**, **i**), 96 h (**b**, **f**, **j**), 7 days (**c**, **g**, **k**) and 14 days (**d**, **h**, **l**). First signs of Cd-induced alterations such as chlorotic lesions could be observed in the mutants 7 days (**g**, **k**) after Cd treatment which became more prominent and covered larger areas 14 days (**h**, **l**) after Cd treatment. Wildtype plants showed first signs of chlorotic lesions starting at 14 days after Cd treatment (**d**)

**Fig. 2:**
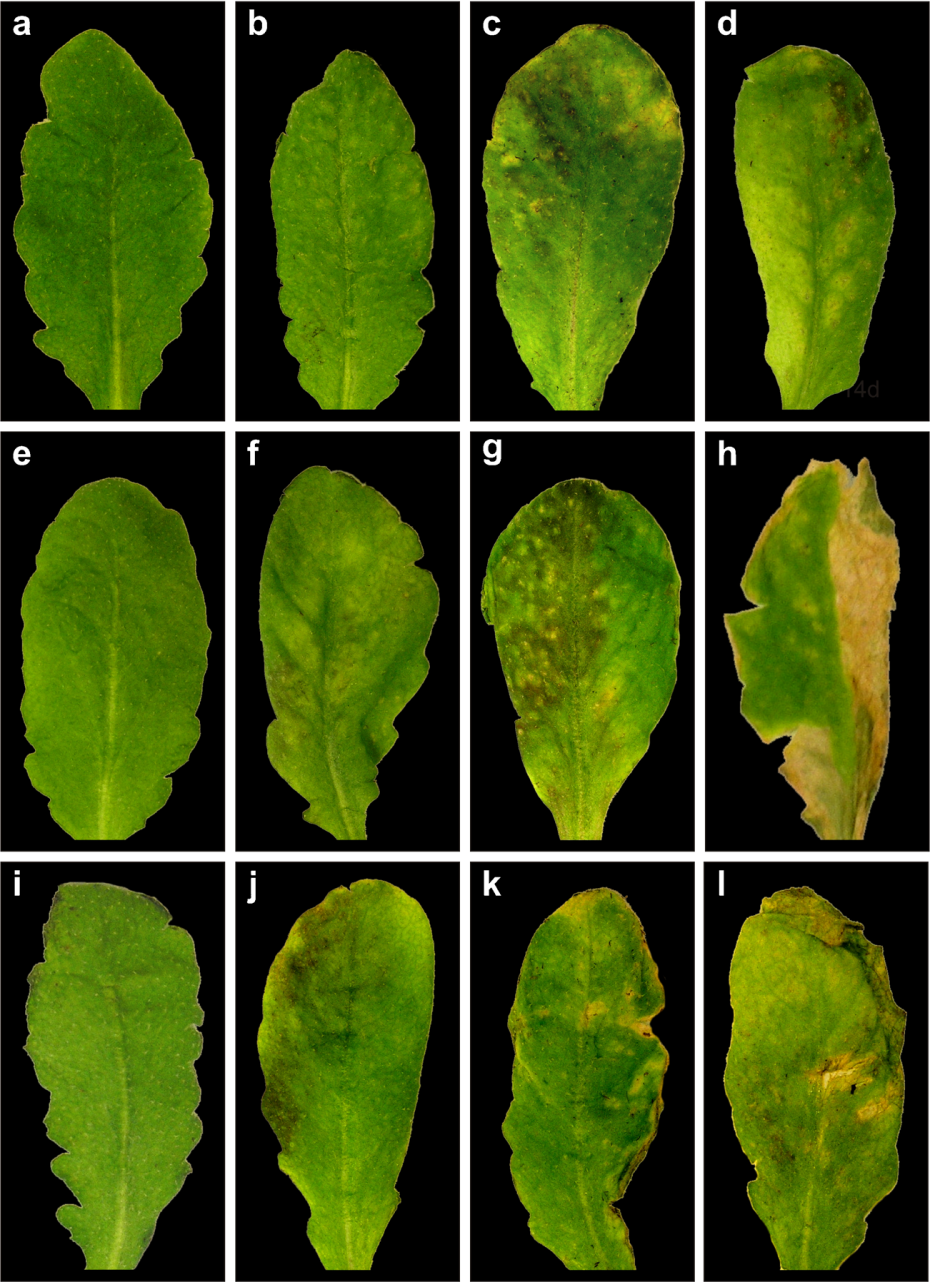
Representative images of leaves from *Arabidopsis thaliana* Col-0 (**a–d**), and the mutants *pad2-1* (**e–h**) and *vtc2-1* (**i–l**). Leaves derived from plants which were treated without Cd for 14 days (**a**, **e**, **i**) or with 100 μM of Cd for 96 h (**b**, **f**, **j**), 7 days (**c**, **g**, **k**) and 14 days (**d**, **h**, **l**). First signs of Cd-induced alterations such as the appearance of chlorosis and necrosis could be observed in the mutants 96 h (**f**, **j**) after Cd treatment which became more prominent and covered larger areas 7 days (**g**, **k**) and 14 days (**h**, **l**) after Cd treatment. Wildtype plants showed first signs of chlorotic lesions starting at 7 days after Cd treatment (**c**) which covered larger areas 14 days after Cd treatment (**d**)

Leaves of plants that were treated without Cd did not develop chlorosis and necrosis (Figs. 1a,e,i and 2a,e,i). First negative effects of Cd treatment in the form of local chlorotic spots could be found on wildtype plants 14 days after the treatment with 50 μM Cd (Fig. 1d). Leaves of *pad2-1* mutants showed first signs of chlorosis and necrosis after 7 days of 50 μM Cd treatment which became more severe 14 days after treatment with 50 μM Cd (Fig. 1g, h.). *vtc2-1* mutants showed minor signs of chlorosis after 7 days and strong chlorotic spots 14 days after the treatment with 50 μM Cd (Fig. 1k, l). After treatment with 100 μM Cd wildtype plants developed chlorosis after 7 days, which became more severe 14 days after Cd treatment (Fig. 2c,d). Leaves of *pad2-1* mutants showed first signs of chlorosis after 96 h (Fig. 2f) and both chlorosis and necrosis after 7 days of 100 μM Cd treatment (Fig. 2g). Large necrotic areas and chlorotic spots were visible on leaves of *pad2-1* mutants 14 days after Cd treatment (Fig. 2h). Leaves of *vtc2-1* mutants showed first signs of chlorosis after 96 h (Fig. 2j) of treatment with 100 μM Cd (Fig. 2j). Chlorosis and necrosis became more severe 7 and 14 days after 100 μM of Cd treatment but did not reach the extent observed in *pad2-1* and Col-0 (Fig. 2k,l).

### Immunogold labeling of ascorbate

#### 50 μM Cd

The subcellular distribution of ascorbate in control plants was similar in most cell compartments to what has been reported previously (Zechmann et al. 2011c). Wildtype plants and *pad2-1* mutants contained similar levels of ascorbate in all cell compartments whereas the *vtc2-1* mutant contained between −50 % and −75 % less ascorbate (vacuoles and nuclei, respectively) than the wildtype (Table 1). These results are similar to what has been described in previous studies which revealed that the *vtc2-1* mutant contained between −50 and −60 % less ascorbate than wildtype plants (Zechmann et al. 2011c).

**Table 1:** Values are means with standard errors and document the total amount of gold particles bound to ascorbate per μm^2^ in different cell compartments of *Arabidopsis thaliana* [L.] Heynh. ecotype Columbia (Col-0), *pad2-1* and *vtc2-1* mutants grown under control conditions

	Col-0	*pad2-1*	*vtc2-1*
Mitochondria	7 ± 0.5^cd^	10 ± 1^b^	2 ± 0.7^hg^
Chloroplasts	6 ± 0.4^d^	6 ± 0.5^d^	3 ± 0.3^ef^
Nuclei	16 ± 0.8^a^	12 ± 0.8^b^	4 ± 0.6^e^
Peroxisomes	10 ± 0.9^b^	12 ± 1^b^	4 ± 0.9^e^
Cytosol	10 ± 0.7^b^	9 ± 0.8^bc^	3 ± 0.6^ef^
Vacuoles	2 ± 0.2^hg^	2 ± 0.1^hg^	1 ± 0.2^g^

*n*>20 for peroxisomes and vacuoles and *n*>60 for other cell structures. Different lowercase letters indicate significant differences (*P* < 0.05) analyzed with the Kruskal–Wallis test followed by post-hoc comparison according to Conover

**Fig. 3:**
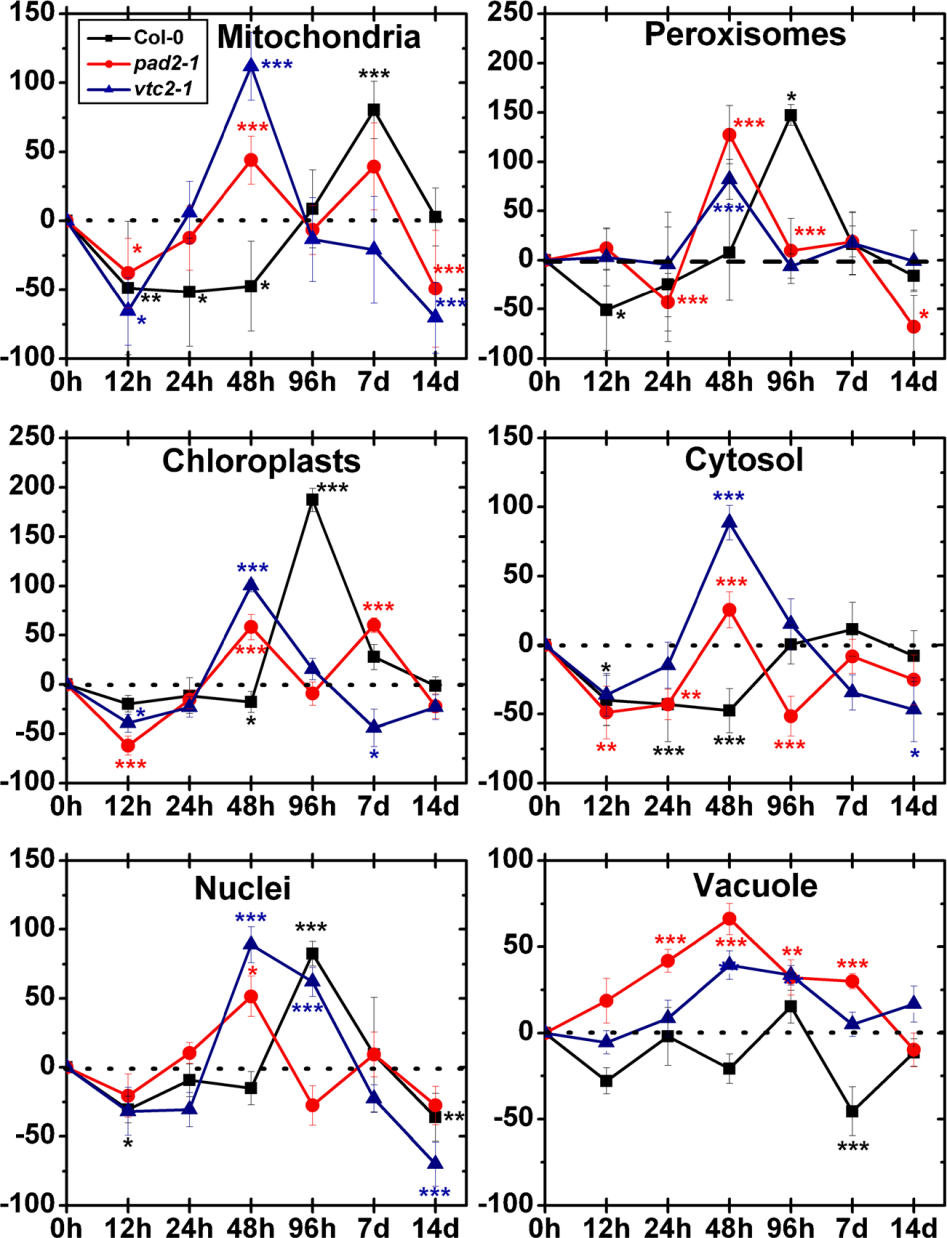
Compartment specific changes in ascorbate labeling density at different time points after the treatment of 50 μM Cd. Graphs show the percentage of increase and decrease of gold particles bound to ascorbate per μm^2^ in mesophyll cells of *Arabidopsis thaliana* Col-0 plants (*black squares*) and the Arabidopsis mutants *pad2-1* (*red circles*) and *vtc2-2* (*blue triangles*). Differences were calculated by comparing Cd-treated samples with control samples which were treated with nutrient solution without Cd (values can be found in Table 1). *n*>20 for peroxisomes and vacuoles and *n*>60 for other cell structures. Data are means with standard errors. Significant differences were calculated using the Mann–Whitney *U*-test; *, ** and *** indicate significance at the 0.05, 0.01 and 0.001 levels of confidence, respectively

Mitochondria of wildtype plants showed significant decreased levels of gold particles bound to ascorbate after 12 h (−49 %), 24 h (−52 %) and 48 h (47 %) and significant increased levels after 7 days (80 %) after treatment with 50 μM Cd (Fig. 3). In chloroplasts decreased amounts of gold particles bound to ascorbate could be observed after 48 h (−18 %), whereas increased ascorbate levels could be observed after 96 h (187 %) of Cd treatment when compared to the control. The amount of gold particles in nuclei significantly decreased after exposure to 50 μM Cd for 12 h (−31 %) and 14 days (−36 %) and showed increased levels after 96 h (82 %) compared to the control. In the cytosol decreased ascorbate levels could be found after 24 h (−43 %) and 48 h (−48 %) after treatment with 50 μM Cd. In peroxisomes significantly decreased ascorbate levels could be observed after 12 h (−51 %) and increased levels after 96 h (147 %). In the vacuole, the numbers of gold particles bound to ascorbate showed a decrease after 7 days (−46 %) whereas no change was found at the other time points when compared to the control (Fig. 3, Fig. S1).

Mitochondria of *pad2-1* mutants showed significantly decreased ascorbate levels after 12 h (−38 %) and 14 days (−49 %) and increased levels after 48 h (44 %) after treatment with 50 μM Cd (Fig. 3). The amount of gold particles bound to ascorbate in chloroplasts significantly decreased after 12 h (−62 %), whereas after 48 h (58 %) and 7 days (60 %) an increase of gold particles compared to the control could be observed. In nuclei, increased amounts of gold particles bound to ascorbate could be observed after 48 h (51 %) after Cd treatment. In the cytosol, the ascorbate levels decreased significantly (between −43 % and −52 %) after 12, 24 and 96 h, whereas after 48 h significantly increased ascorbate levels (26 %) could be observed. The amount of gold particles bound to ascorbate in peroxisomes significantly increased after exposure to Cd for 12 h (12 %), 48 h (127 %) and 96 h (10 %) and decreased after 24 h (43 %) and 14 days (68 %). The vacuole showed significant increased ascorbate levels after Cd treatment for 24 h (42 %), 48 h (66 %), 96 h (32 %) and 7 days (30 %) compared to the control (Fig. 3, Fig. S2).

In the mitochondria of *vtc2-1* mutants, the labeling density was significantly increased after 48 h (112 %), whereas decreased levels of ascorbate were found after 12 h (−36 %) and 14 days (−70 %) after treatment with 50 μM Cd (Fig. 3). In chloroplasts, the number of gold particles bound to ascorbate was significantly decreased after 12 h (−39 %) and 7 days (44 %) whereas after 48 h an increase of 100 % could be observed. The amount of gold particles bound to ascorbate in nuclei was significantly increased after 48 h (89 %) and 96 h (62 %) whereas decreased levels could be observed after 14 days (−70 %) after treatment with 50 μM Cd. In the cytosol ascorbate levels significantly decreased after 12 h (−36 %) and 14 days (−47 %) and increased after 48 h (89 %) compared to the control. Generally the ascorbate levels in peroxisomes were similar to control levels except after 48 h after Cd treatment where an increase of 82 % could be observed. The vacuole showed increased ascorbate levels after 48 h (39 %) and 96 h (33 %) compared to the control (Fig. 3, Fig. S3).

#### 100 μM Cd

**Fig. 4:**
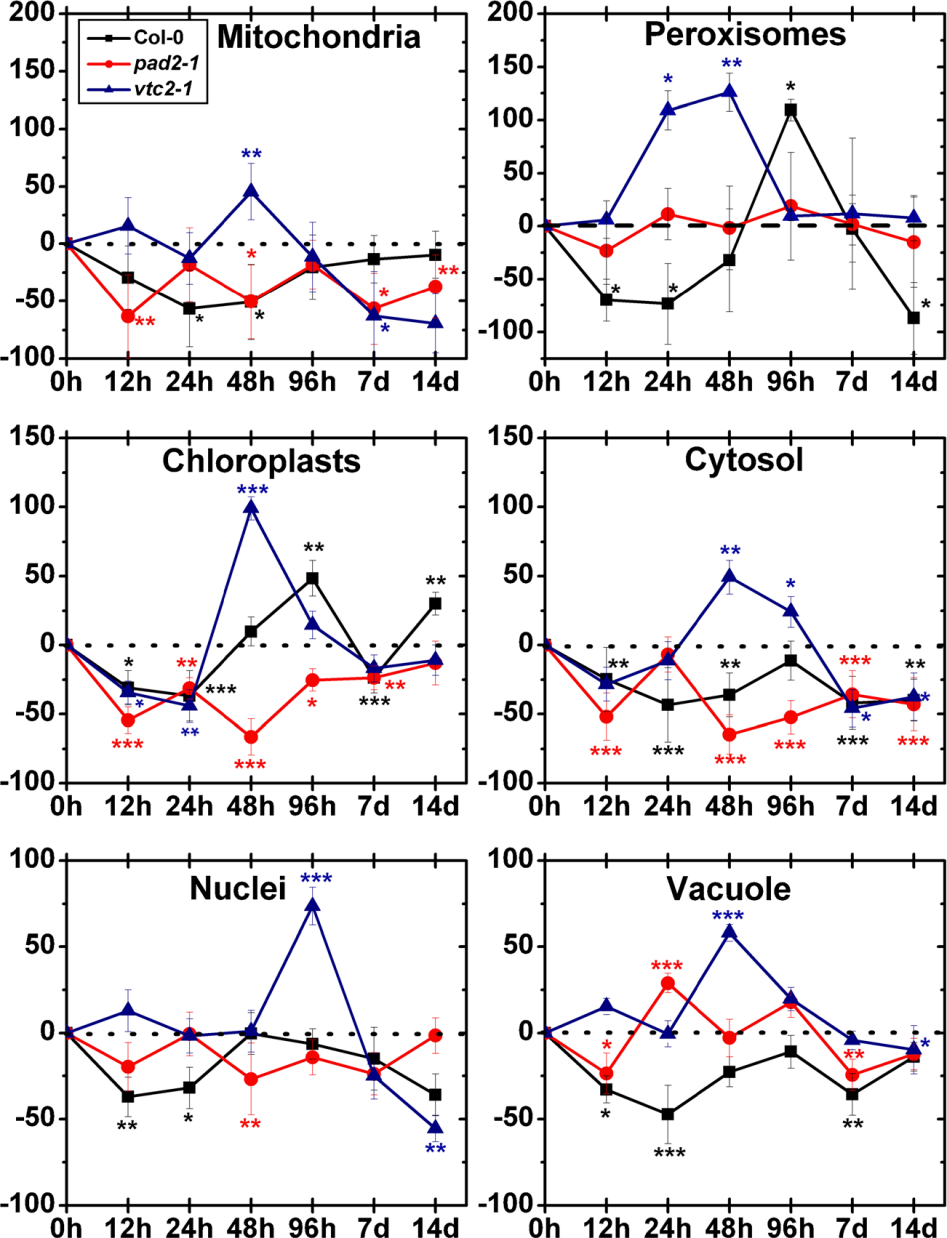
Compartment specific changes in ascorbate labeling density at different time points after the treatment of 100 μM Cd. Graphs show the percentage of increase and decrease of gold particles bound to ascorbate per μm^2^ in mesophyll cells of *Arabidopsis thaliana* Col-0 plants (*black squares*) and the Arabidopsis mutants *pad*2-1 (*red circles*) and *vtc2-2* (*blue triangles*). Differences were calculated by comparing Cd-treated samples with control samples which were treated with nutrient solution without Cd (values can be found in Table 1). *n*>20 for peroxisomes and vacuoles and *n*>60 for other cell structures. Data are means with standard errors. Significant differences were calculated using the Mann–Whitney *U*-test; *, ** and *** indicate significance at the 0.05, 0.01 and 0.001 levels of confidence, respectively

In the mitochondria of wildtype plants, the labeling density of ascorbate was significantly decreased after 24 h (−42 %) and 48 h (−51 %) after treatment with 100 μM Cd (Fig. 4). In plastids decreased amounts of gold particles bound to ascorbate could be observed after 12 h (−31 %), 24 h (−37 %) and 7 days (−24 %), whereas significant increased levels was found after 96 h (48 %) and 14 days (30 %) when compared to the control. In nuclei, the gold particle density bound to ascorbate decreased significantly after 12 h (−37 %) and 24 h (−32 %). Generally, the ascorbate levels in the cytosol where significantly decreased (up to −43 %). In peroxisomes, the numbers of gold particles bound to ascorbate increased after 96 h (109 %) compared to the control whereas after 12 h (−50 %), 24 h (−47 %) and 14 days (−64 %) significant decreased ascorbate levels could be observed. The vacuole showed decreased levels of ascorbate after 12 h (−33 %), 24 h (47 %) and 7 days (−36 %) after treatment with 100 μM Cd (Fig. 4, Fig. S1).

Mitochondria of *pad2-1* mutants showed after 12 h (−63 %), 48 h (−43 %), 7 days (−36 %) and 14 days (−37 %) significant decreased levels of ascorbate after treatment with 100 μM Cd (Fig. 4). In plastids, the ascorbate levels were generally decreased (between −13 % and −66 %) when compared to the control. In nuclei, significant decreased amounts of gold particles bound to ascorbate could be observed after 48 h (−27 %). In the cytosol, the numbers of gold particles where decreased significantly after 12 h, 48 h, 96 h, 7 days and 14 days (between −36 % and −65 %). In peroxisomes, the labeling density of ascorbate showed no significant differences to the control. In the vacuole, significant decreased ascorbate levels could be observed after 12 h (−23 %) and 7 days (24 %), whereas after 24 h the number of gold particles bound to ascorbate increased up to 29 % (Fig. 4, Fig. S2).

After exposure to 100 μM Cd, mitochondria of *vtc2-1* mutants showed a significant increase of ascorbate levels after 48 h (45 %) whereas after 7 days (−51 %) and 14 days (−54 %) significant decreased levels compared to the control could be observed (Fig. 4). In plastids the amounts of gold particles bound to ascorbate significantly decreased after 12 h (−34 %) and 24 h (−44 %), whereas after 48 h increased ascorbate levels compared to the control could be observed (99 %). In nuclei the labeling density of ascorbate was significantly increased after 96 h (73 %), whereas decreased levels were found after 14 days (−56 %). In the cytosol the numbers of gold particles bound to ascorbate significantly increased after 48 h (49 %) and 96 h (24 %), whereas after 7 days (−46 %) and 14 days (−37 %) decreased ascorbate levels could be observed. In peroxisomes the labeling density of ascorbate increased significantly after 24 h (109 %) and 48 h (126 %) compared to the control. In the vacuole a significant higher labeling density of ascorbate could be observed after 48 h (58 %) compared to the control (Fig. 4, Fig.S3).

### Immunogold labeling of glutathione

#### 50 μM Cd

The subcellular distribution of glutathione in control plants was similar in most cell compartments to what has been reported previously (Zechmann and Müller 2010). Wildtype plants and *vtc2-1* mutants contained similar levels of glutathione in all cell compartments whereas the *pad2-1* mutant contained between −87 % and −71 % less glutathione (nuclei and chloroplasts, respectively) than the wildtype (Table 2). In mitochondria, the *pad2-1* mutant contained similar levels of glutathione when compared to the wildtype and the *vtc2-1* mutant (Table 2). These results are similar to what has been described in previous studies which revealed that mitochondria of the *pad2-1* mutant contained wildtype glutathione levels whereas all other cell compartments contained up to −90 % less glutathione than the wildtype (Zechmann et al. 2008). Additionally, it has been shown in previous studies that *vtc2-1* mutants contain similar glutathione levels when compared to the wildtype (Fernandez-Garcia et al. 2009).

**Table 2:** Values are means with standard errors and document the total amount of gold particles bound to glutathione per μm^2^ in different cell compartments of *Arabidopsis thaliana* [L.] Heynh. ecotype Columbia (Col-0), *pad2-1* and *vtc2-1* mutants grown under control conditions

	Col-0	*pad2-1*	*vtc2-1*
Mitochondria	302 ± 30^a^	286 ± 46^a^	240 ± 15^ab^
Chloroplasts	35 ± 5^f^	10 ± 1^j^	25 ± 3^gi^
Nuclei	209 ± 34^bc^	28 ± 2^g^	160 ± 13^cd^
Peroxisomes	109 ± 12^e^	20 ± 1^hi^	82 ± 10^e^
Cytosol	130 ± 17^de^	20 ± 0.8^g^	140 ± 15^d^

*n*>20 for peroxisomes and vacuoles and *n*>60 for other cell structures. Different lowercase letters indicate significant differences (*P* < 0.05) analyzed with the Kruskal–Wallis test followed by post-hoc comparison according to Conover

**Fig. 5:**
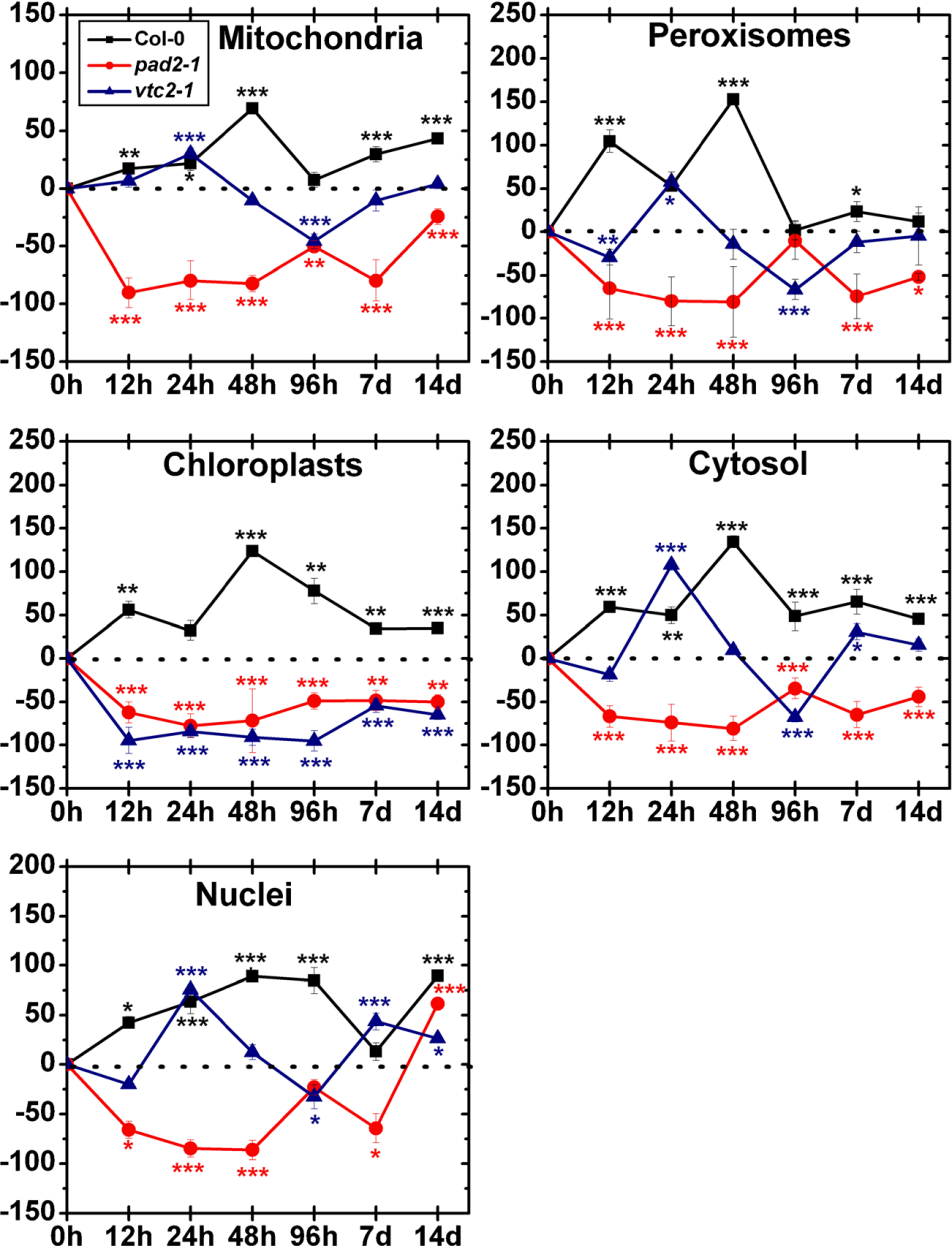
Compartment specific changes in glutathione labeling density at different time points after the treatment of 50 μM Cd. Graphs show the percentage of increase and decrease of gold particles bound to glutathione per μm^2^ in mesophyll cells of *Arabidopsis thaliana* Col-0 plants (*black squares*) and the Arabidopsis mutants *pad2-1* (*red circles*) and *vtc2-2* (*blue triangles*). Differences were calculated by comparing Cd-treated samples with control samples which were treated with nutrient solution without Cd (values can be found in Table 2). *n*>20 for peroxisomes and vacuoles and *n*>60 for other cell structures. Data are means with standard errors. Significant differences were calculated using the Mann–Whitney *U*-test; *, ** and *** indicate significance at the 0.05, 0.01 and 0.001 levels of confidence, respectively

Mitochondria of wildtype plants showed a significant increase of gold particles bound to glutathione after 12 h (17 %), 24 h (22 %), 48 h (69 %), 7 days (30 %) and 14 days (43 %) treatment with 50 μM Cd (Fig. 5). In chloroplasts, elevated glutathione levels between 32 % and 124 % compared to the control could be observed at all time points. In nuclei, increased amounts of gold particles bound to glutathione could be observed after 12 h (42 %), 24 h (63 %), 48 h (89 %), 96 h (84 %) and 14 days (90 %) compared to the control. In the cytosol increased glutathione levels were found over the whole experiment (between 45 % and 134 %) after exposure to 50 μM Cd. The amount of gold particles bound to glutathione in peroxisomes significantly increased after exposure to Cd for 12 h (104 %), 24 h (53 %), 48 h (153 %) and 7 days (23 %; Fig. 5, Fig. S4).

After exposure to 50 μM Cd, mitochondria (between −24 % and −90 %), chloroplasts (between −48 % and −78 %) and the cytosol (between −34 % and −81 %) of *pad2-1* mutants showed significantly decreased glutathione levels at all time points when compared to the control (Fig. 5). Generally, glutathione labeling in nuclei was significantly decreased (between −23 % and −86 %) until 14 days, where surprisingly a significant increase of 62 % could be observed (Fig. 5, Fig. S5). Peroxisomes showed significantly decreased amounts of gold particles bound to glutathione after Cd treatment for 12 h (−65 %), 24 h (−80 %), 48 h (−81 %), 7 days (−74 %) and 14 days (−52 %).

In mitochondria of *vtc2-1* mutants the labeling density was significantly increased after 24 h (30 %), whereas decreased levels were found after 96 h (−46 %) of Cd treatment (Fig. 5). No change was found at the other time points. In chloroplasts the labeling density of glutathione significantly decreased (between −54 % and −95 %) over the whole experiment when compared to the control. Nuclei of *vtc2-1* mutants showed significantly higher amounts of gold particles bound to glutathione after Cd treatment for 24 h (75 %), 7 days (43 %) and 14 days (26 %) whereas decreased levels were found after 96 h (−33 %). In cytosol, increased amounts of gold particles bound to glutathione could be observed after 24 h (107 %) and 7 days (31 %) compared to the control whereas a significant decrease of −68 % could be analyzed after Cd treatment for 96 h. In peroxisomes, the gold particle density decreased −29 % and −66 % after Cd treatment for 12 h and 96 h whereas after 24 h the number of gold particles bound to glutathione increased up to 58 % compared to the control (Fig. 5, Fig. S6).

#### 100 μM Cd

**Fig. 6:**
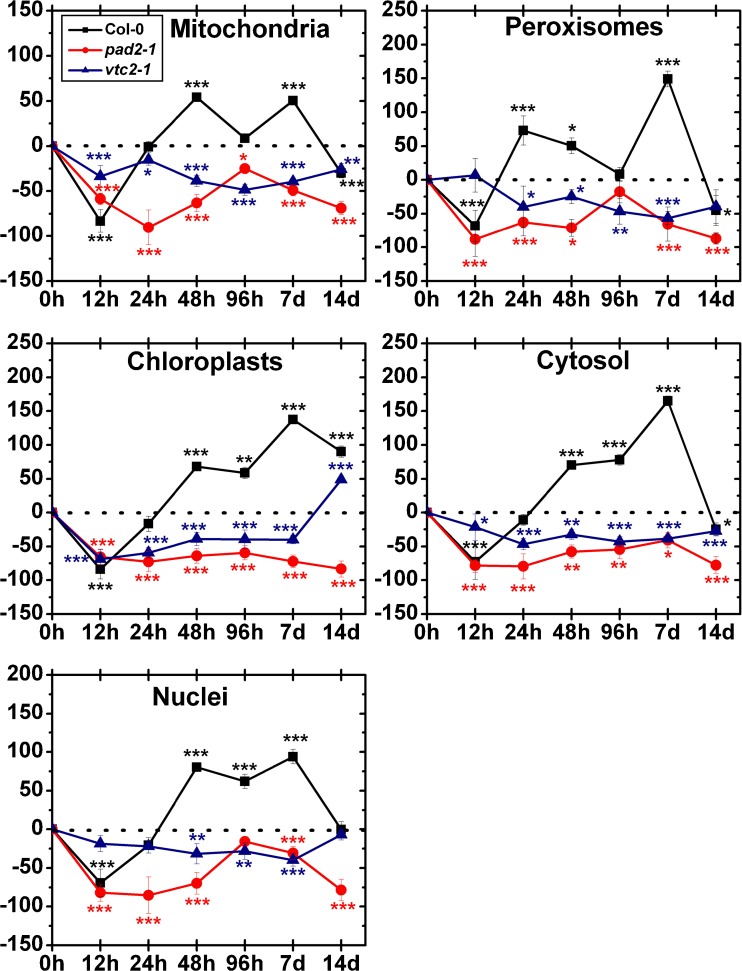
Compartment specific changes in glutathione labeling density at different time points after the treatment of 100 μM Cd. Graphs show the percentage of increase and decrease of gold particles bound to glutathione per μm^2^ in mesophyll cells of *Arabidopsis thaliana* Col-0 plants (*black squares*) and the Arabidopsis mutants *pad2-1* (*red circles*) and *vtc2-2* (*blue triangles*). Differences were calculated by comparing Cd-treated samples with control samples which were treated with nutrient solution without Cd (values can be found in Table 2). *n*>20 for peroxisomes and vacuoles and *n*>60 for other cell structures. Data are means with standard errors. Significant differences were calculated using the Mann–Whitney *U*-test; *, ** and *** indicate significance at the 0.05, 0.01 and 0.001 levels of confidence, respectively

Mitochondria of wildtype plants showed significantly higher amounts of gold particles bound to glutathione after treatment with 100 μM Cd for 48 h (54 %) and 7 days (50 %) whereas decreased levels were found after 12 h (−83 %) and 14 days (−30 %, Fig. 6). In chloroplasts, significantly decreased glutathione levels could be observed after 12 h (−84 %) and increased levels after 48 h (68 %), 96 h (58 %), 7 days (137 %) and 14 days (90 %) when compared to the control. After 12 h the gold particle density in nuclei was significantly decreased (−69 %) whereas after 48 h, 96 h and 7 days an increase between 62 % and 94 % compared to the control could be observed. The cytosol showed significantly decreased amounts of gold particles bound to glutathione after Cd treatment for 12 h (−73 %) and 14 days (−25 %) and increased amounts after 48 h (70 %), 96 h (78 %) and 7 days (165 %) compared to the control. Twelve hours and 14 days after exposure to Cd, the labeling density in peroxisomes significantly decreased (between 45 % and 68 %), while after 24 h, 48 h and 7 days increased amounts of gold particles (between 50 % and 149 %) could be observed (Fig. 6, Fig. S4).

Significantly decreased glutathione labeling could be observed in mitochondria (between −25 % and −90 %), chloroplasts (between −59 % and −83 %) and cytosol (between −41 % and 80 %) of *pad2-1* mutants at all time points after exposure to 100 μM Cd. In nuclei and peroxisomes, the numbers of gold particles bound to glutathione were generally decreased (between −31 % and −85 % in nuclei and between −63 % and −88 % in peroxisomes), and no change was found after 96 h compared to the control (Fig. 6, Fig. S5).

At all time points the exposure to Cd significantly decreased labeling density in mitochondria (between −16 % and −49 %) and the cytosol (between −22 % and −47 %) in *vtc2-1* mutants. Generally, glutathione labeling in chloroplasts was significantly decreased for the first 7 days (between −39 % and −69 %) whereas 14 days after Cd exposure an increase of 49 % compared to the control could be observed. The amount of gold particles bound to glutathione in nuclei significantly decreased after exposure to Cd for 48 h (−32 %), 96 h (−28 %) and 7 days (−40 %). In peroxisomes significantly decreased glutathione levels (between 40 % and 56 %) were found after the exposure to Cd for 24 h, 96 h, 7 days and 14 days (Fig. 6; Fig. S6).

## Discussion

In general, *pad2-1* and *vtc2-1* mutants were more sensitive to the exposure to Cd than wildtype plants. After the exposure to Cd both mutants showed symptoms earlier than the wildtype and developed stronger signs of chlorosis and necrosis which covered larger areas of the leaves. *pad2-1* mutants which contain up to 90 % less glutathione than wildtype plants (Parisy et al. 2006; Zechmann et al. 2008) showed even higher sensitivity to Cd exposure than *vtc2-1* mutants as symptoms occurred earlier and stronger than in *vtc2-1* mutants. Throughout the experiment, the higher sensitivity of *pad2-1* and *vtc2-1* mutants could be correlated to much lower glutathione contents when compared to Col-0, which indicates the importance of glutathione for the protection of plants against Cd stress. Similar results as those observed in this study have also been obtained for the glutathione deficient *cad2-1* mutant and plants with artificially reduced glutathione contents which also showed higher Cd sensitivity due to low glutathione contents (Howden et al. 1995; Wójcik and Tukiendorf 2011). It was also shown that increased glutathione contents in the *cdr3-1D* mutants are partially required for enhanced Cd resistance (Wang et al. 2011). Even though higher Cd sensitivity of ascorbate deficient mutants has not been documented yet in the literature, such effects seem likely as several physiological studies demonstrated a correlation between low tissue concentrations of ascorbate and glutathione and Cd sensitivity (Wójcik and Tukiendorf 2011). Thus, we can conclude that higher Cd sensitivity of glutathione and ascorbate deficient mutants are due to lower antioxidative capacity of these plants when compared to wildtype plants.

**Fig. 7:**
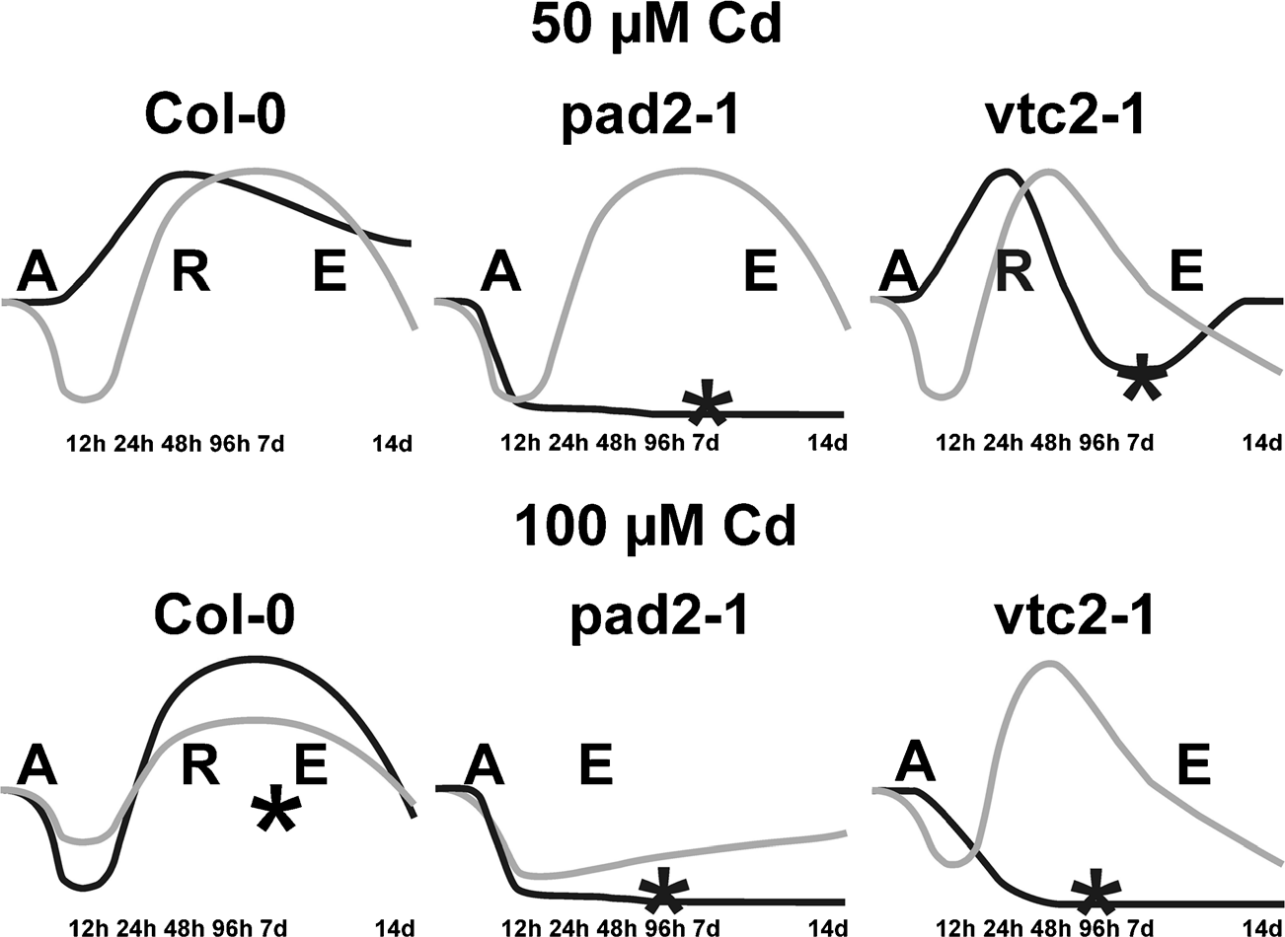
Line drawing showing a response curve of the overall ascorbate and glutathione labeling of Col-0, *pad2-1*, and *vtc2-1* to treatment with different concentrations of Cd. The relative ascorbate and glutathione content was obtained according to Koffler et al. (2013) using the corresponding labeling density during Cd treatment and the relative compartment volume from the leaf center, where the sum runs over all compartments. The alarming phase is indicated with the letter "A", the resistance phase is indicated with the letter "R", and the exhaustion phase is indicated with the letter "E". *Black line* glutathione, *grey line* ascorbate, *asterisk* appearance of symptoms

In this respect, it is interesting that glutathione contents in all cell compartments of wildtype plants followed recently proposed antioxidative stress models (Tausz et al. 2004; Kranner et al. 2010) which correlated well with symptom development on the leaves (Fig. 7). In wildtype plants exposed to 50 μM Cd which showed only minor symptoms 14 days after treatment, glutathione contents increased immediately after Cd exposure and remained at high levels until the end of the experiment indicating an acclimation effect of these plants to the exposure to Cd similar to what was described by Tausz et al. (2004). Ascorbate contents reacted in these plants with an initial decrease in ascorbate contents (initial alarming phase), a strong increase in ascorbate contents (resistance phase) and a strong decrease in ascorbate contents (final exhaustion phase; Fig. 7). As strong symptoms remain minor or absent in these plants, these results indicate that high glutathione contents rather than high ascorbate protect plants from Cd induced symptoms in the long term. Wildtype plants exposed to 100 μM showed a typical bell shaped stress response curve (Kranner et al. 2010) with an immediate decrease in glutathione and ascorbate contents which indicates an excessive demand for antioxidants immediately after the exposure to Cd (Fig. 7). Such a reaction of plants to excess Cd seems likely as Cd has a high affinity to thiol groups and would bind to reduced glutathione present in the cytosol after entering the cells (Semane et al. 2007; DalCorso et al. 2008; Jozefczak et al. 2012). Additionally, glutathione is used for the increased production of PCs which are also involved in the detoxification of Cd (DalCorso et al. 2008; Jozefczak et al. 2012). On top both antioxidants play important roles to prevent the accumulation of ROS which is commonly observed during the exposure of plants to heavy metals (Gill and Tuteja 2010; Yadav 2010). Nevertheless, considering that Cd enters the cytosol first and is not relocated into other cell compartments except vacuoles (Van Belleghem et al. 2007) it is interesting that glutathione and ascorbate decreased in all other cell compartments at similar rates. These results are similar to what we have observed for glutathione contents in previous studies (Kolb et al. 2010) and indicates that glutathione and ascorbate are either withdrawn from the other cell compartments to assuage the higher demand of glutathione in the cytosol or that less freshly produced ascorbate and glutathione get transported from the origin of synthesis (e.g., mitochondria for ascorbate [Bartoli et al. 2000; Millar et al. 2003], plastids and cytosol for glutathione [Wachter et al. 2005]) to the other cell compartments as they are used for Cd and ROS detoxification in the cytosol. This initial phase is then followed by a strong accumulation of glutathione and ascorbate (Fig. 7) which indicates an acclimation or resistance phase where the plant is successfully coping with the environmental stress condition (Tausz et al. 2004; Kranner et al. 2010). Nevertheless, wildtype plants exposed to 100 μM Cd obviously could not withstand Cd stress successfully in the long term as symptoms such as chlorosis and necrotic spots across the leaves could be observed 14 days after Cd exposure. These symptoms could be correlated with a strong decrease in glutathione contents in most cell compartments at this time point. Additionally, ascorbate contents remained at or below control levels in most cell compartments (Fig. 7) indicating that these plants went through an exhaustion phase which is characterized by failure of protection and repair mechanisms (Kranner et al. 2010) and led to strong symptoms such as the appearance of chlorosis and necrotic spots.

Glutathione contents in *pad2-1* mutants which showed highest sensitivity to Cd were in general decreased in all cell compartments throughout the treatment with 50 and 100 μM Cd (Fig. 7). This strong decrease was correlated with strong symptom development early after Cd treatment (e.g., 96 h after treatment with 100 μM Cd) indicating that low glutathione levels in this mutant which contains up to 90 % less glutathione than wildtype plants (Parisy et al. 2006; Zechmann et al. 2008) were unable to protect plants against the toxic effects of Cd (Fig. 7). Similar effects have also been found in plants with artificially decreased glutathione contents and for the glutathione deficient mutant *cad2-1* which showed higher Cd sensitivity correlated with low glutathione contents (Howden et al. 1995; Wójcik and Tukiendorf 2011). Considering that *pad2-1* mutants showed the highest sensitivity to Cd, it becomes obvious that the accumulation of ascorbate in *pad2-1* mutants treated with 50 μM Cd were not able to compensate low glutathione contents in order to prevent symptom development induced by the accumulation of ROS commonly observed during Cd treatment (Gill and Tuteja 2010; Yadav 2010). On the subcellular level, it is interesting that higher Cd sensitivity correlated with a strong reduction of glutathione in mitochondria. The *pad2-1* mutant is characterized by low glutathione contents in all cell compartments (up to 90 % less than Col-0) and wildtype glutathione levels in mitochondria. Additionally, we have recently demonstrated that high and stable levels of glutathione in mitochondria of the *pad2-1* mutants are important for proper plant development (Zechmann and Müller 2010). Thus, the strong decrease of glutathione contents in mitochondria observed in this study of up to 90 % in this mutant could be a key factor for higher Cd sensitivity.

*vtc2-1* mutants reacted to treatment of 50 μM Cd with a bell-shaped stress–response curve (Kranner et al. 2010) in most cell compartments with an initial decrease of glutathione and ascorbate followed by a strong accumulation of both antioxidants and a final drop to or below control values (Fig. 7). *vtc2-1* mutants treated with 100 μM Cd showed a strong decrease of glutathione contents similar to the *pad2-1* mutants but reacted with a strong increase of ascorbate contents after 48 days. Nevertheless, similar to the *pad2-1* mutants, a strong decrease of both antioxidants could be observed in the long term at 100 μM Cd treatment which correlates well with symptom development such as chlorosis and necrosis on the leaves. In this respect it is remarkable that the *vtc2-1* mutants which have impaired ascorbate synthesis leading to 60 % less ascorbate in the tissue when compared to Col-0 (Conklin et al. 2000; Zechmann et al. 2011c) reacted with a strong increase in ascorbate contents upon Cd treatment (observed mainly at 48 and 96 h after treatment). Similar results have also been observed during exposure to high light where *vtc2-1* mutants surprisingly reacted with an increase in ascorbate contents despite distorted ascorbate synthesis (Müller-Moulé et al. 2004).

On the subcellular level, it is interesting that gold particles bound to glutathione could not be detected in vacuoles even in Cd-treated samples. It is commonly accepted that glutathione forms complexes with Cd through the thiol groups which are then sequestered in vacuoles (Rauser 2001; Maksymiec and Krupa 2006; Semane et al. 2007; DalCorso et al. 2008; Jozefczak et al. 2012). Thus, the lack of labeling in vacuoles in Cd-treated samples indicates that the glutathione antibody method used in this study detects free glutathione only. These results are supported by previous labeling experiments which showed the lack of labeling in vacuoles of Arabidopsis leaf disks treated with monochlorobimane which are similar to Cd bound to the thiol groups of reduced glutathione and then transported into vacuoles (Queval et al. 2011). Long-term exposure to Cd (14 days) induced the depletion of glutathione and ascorbate contents in all cell compartments except chloroplasts. These results indicate an important role for antioxidants in chloroplasts for the protection of plants against Cd. It is well known that Cd inhibits photosynthesis as it disturbs the synthesis of chlorophyll and carotenoids, inhibits the enzyme activity of the Calvin cycle and leads to CO_2_ deficiency due to stomatal closure, which cause the inhibition of the photosynthesis (Ding et al. 2006). As glutathione and ascorbate decreased in all other cell compartments, these results indicate that the plant accumulated antioxidants in chloroplasts at the expense of ascorbate and glutathione of the other cell compartments. This is especially interesting as the cytosol is supposed to be the primary cell compartment for Cd detoxification as it is not distributed to other cell compartments except vacuoles (Van Belleghem et al. 2007). Thus, the long-term accumulation of ascorbate and glutathione in chloroplasts indicates that the plant redistributes glutathione and ascorbate into chloroplasts during long-term Cd exposure most probably to detoxify ROS produced during photosynthesis.

## Conclusion

Summing up, we can conclude that ascorbate and glutathione deficient mutants showed higher sensitivity to Cd. Nevertheless, glutathione deficient *pad2-1* mutants showed even higher sensitivity to Cd and reacted to Cd with a strong decrease in glutathione contents similar to *vtc2-1* mutants. As symptom development seemed to be related with low glutathione contents rather than low ascorbate contents (Fig. 7), it seems that low glutathione contents rather than low ascorbate contents are responsible for the high Cd sensitivity in these plants.

## Electronic supplementary material

Below is the link to the electronic supplementary material.

Fig.S1 Representative transmission electron micrographs showing gold particles bound to ascorbate on leaf sections from *Arabidopsis thaliana* Col-0. Plants were treated with 0 (**a**), 50 (**b–d**), and 100 μM Cd (**e–g**) for 12 h (**b**, **e**), 48 h (**c**, **f**), and 14 days (**a**, **d**, **g**). Bars = 0.5 μm. *C* chloroplasts with or without starch (*St*), *CW* cell walls, *M* mitochondria, *N* nuclei, *Px* peroxisomes, *V* vacuoles (JPEG 5313 kb)

Fig. S2 Representative transmission electron micrographs showing gold particles bound to ascorbate on leaf sections from *Arabidopsis thaliana pad2-1*. Plants were treated with 0 (**a**), 50 (**b–d**), and 100 μM Cd (**e–g**) for 12 h (**b**, **e**), 96 h (**c**, **f**), and 14 days (**a**, **d**, **g**). Bars = 0.5 μm. *C* chloroplasts with or without starch (*St*), *CW* cell walls, *M* mitochondria, *N* nuclei, *Px* peroxisomes, *V* vacuoles (JPEG 5448 kb)

Fig. S3 Representative transmission electron micrographs showing gold particles bound to ascorbate on leaf sections from *Arabidopsis thaliana vtc2-1*. Plants were treated with 0 (**a**), 50 (**b–d**), and 100 μM Cd (**e–g**) for 12 h (**b**, **e**), 48 h (**c**, **f**), and 14 days (**a**, **d**, **g**). Bars = 0.5 μm. *C* chloroplasts with or without starch (*St*), *CW* cell walls, *M* mitochondria, *N* nuclei, *Px* peroxisomes, *V* vacuoles (JPEG 5514 kb)

Fig. S4 Representative transmission electron micrographs showing gold particles bound to glutathione on leaf sections from *Arabidopsis thaliana* Col-0. Plants were treated with 0 (**a**), 50 (**b–d**), and 100 μM Cd (**e–g**) for 12 h (**b**, **e**), 48 h (**c**, **f**), and 14 days (**a**, **d**, **g**). Bars = 0.5 μm. *C* chloroplasts with or without starch (*St*), *CW* cell walls, *M* mitochondria, *N* nuclei, *Px* peroxisomes, *V* vacuoles (JPEG 5347 kb)

Fig. S5 Representative transmission electron micrographs showing gold particles bound to glutathione on leaf sections from *Arabidopsis thaliana pad2-1*. Plants were treated with 0 (**a**), 50 (**b–d**), and 100 μM Cd (**e–g**) for 12 h (**b**, **e**), 96 h (**c**, **f**), and 14 days (**a**, **d**, **g**). Bars = 0.5 μm. *C* chloroplasts with or without starch (*St*), *CW* cell walls, *M* mitochondria, *N* nuclei, *Px* peroxisomes, *V* vacuoles (JPEG 5448 kb)

Fig. S6 Representative transmission electron micrographs showing gold particles bound to glutathione on leaf sections from *Arabidopsis thaliana vtc2-1*. Plants were treated with 0 (**a**), 50 (**b–d**), and 100 μM Cd (**e–g**) for 12 h (**b**, **e**), 48 h (**c**, **f**), and 14 days (**a**, **d**, **g**). Bars = 0.5 μm. *C* chloroplasts with or without starch (*St*), *CW* cell walls, *M* mitochondria, *N* nuclei, *Px* peroxisomes, *V* vacuoles (JPEG 5246 kb)
