# Supplementary figures and images for: Higher sensitivity of pad2-1 and vtc2-1 mutants to cadmium is related to lower subcellular glutathione rather than ascorbate contents
Source: Protoplasma. 2013 Nov 27;251(4):755–69. doi: 10.1007/s00709-013-0576-x (PMC4059996; doi:10.1007/s00709-013-0576-x)

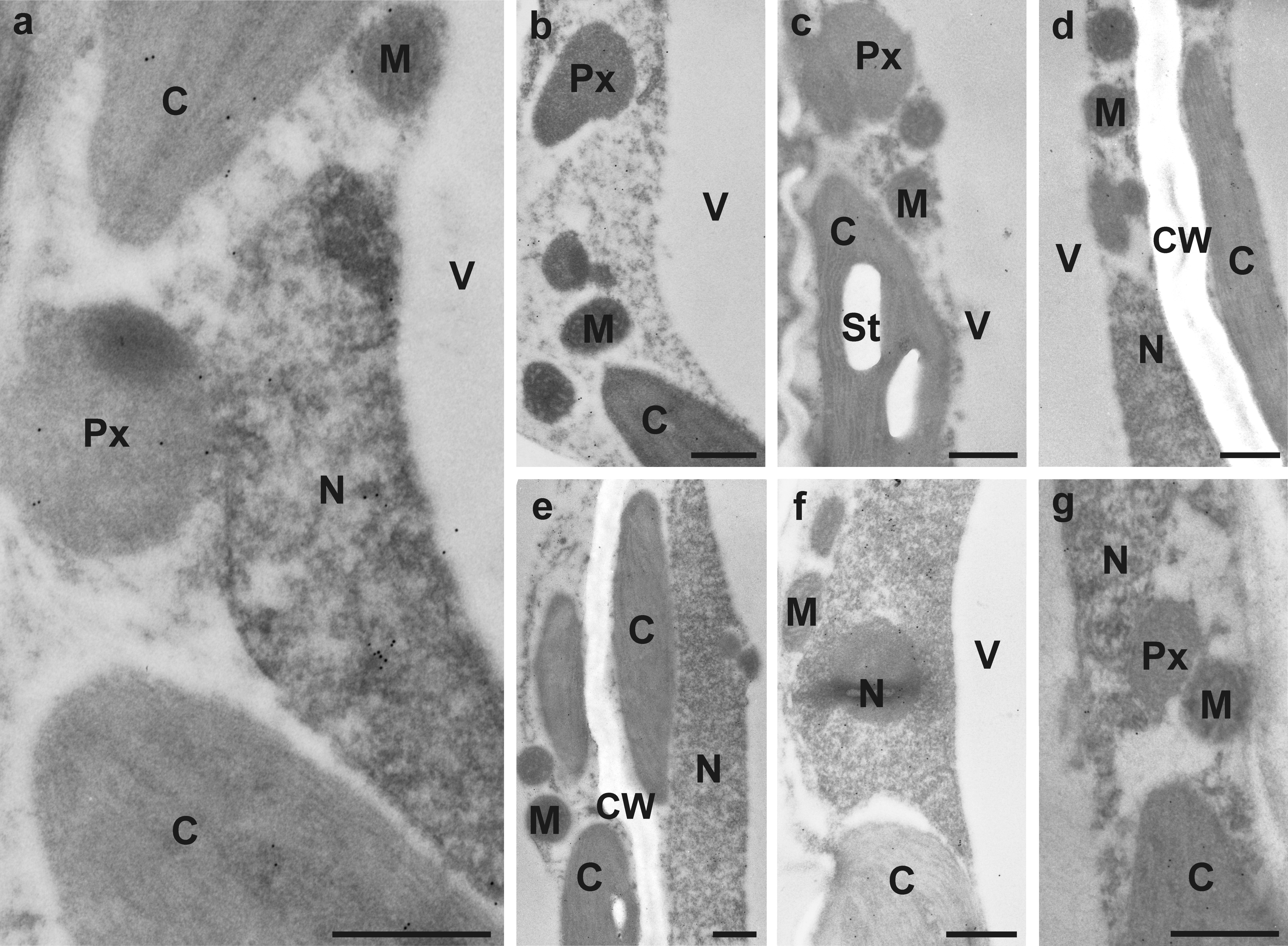

Supplement: Supplementary file 1 — Representative transmission electron micrographs showing gold particles bound to ascorbate on leaf sections from Arabidopsis thaliana Col-0. Plants were treated with 0 (a), 50 (b–d), and 100 μM Cd (e–g) for 12 h (b, e), 48 h (c, f), and 14 days (a, d, g). Bars = 0.5 μm. C chloroplasts with or without starch (St), CW cell walls, M mitochondria, N nuclei, Px peroxisomes, V vacuoles (JPEG 5313 kb) [file 709_2013_576_MOESM1_ESM.jpg]

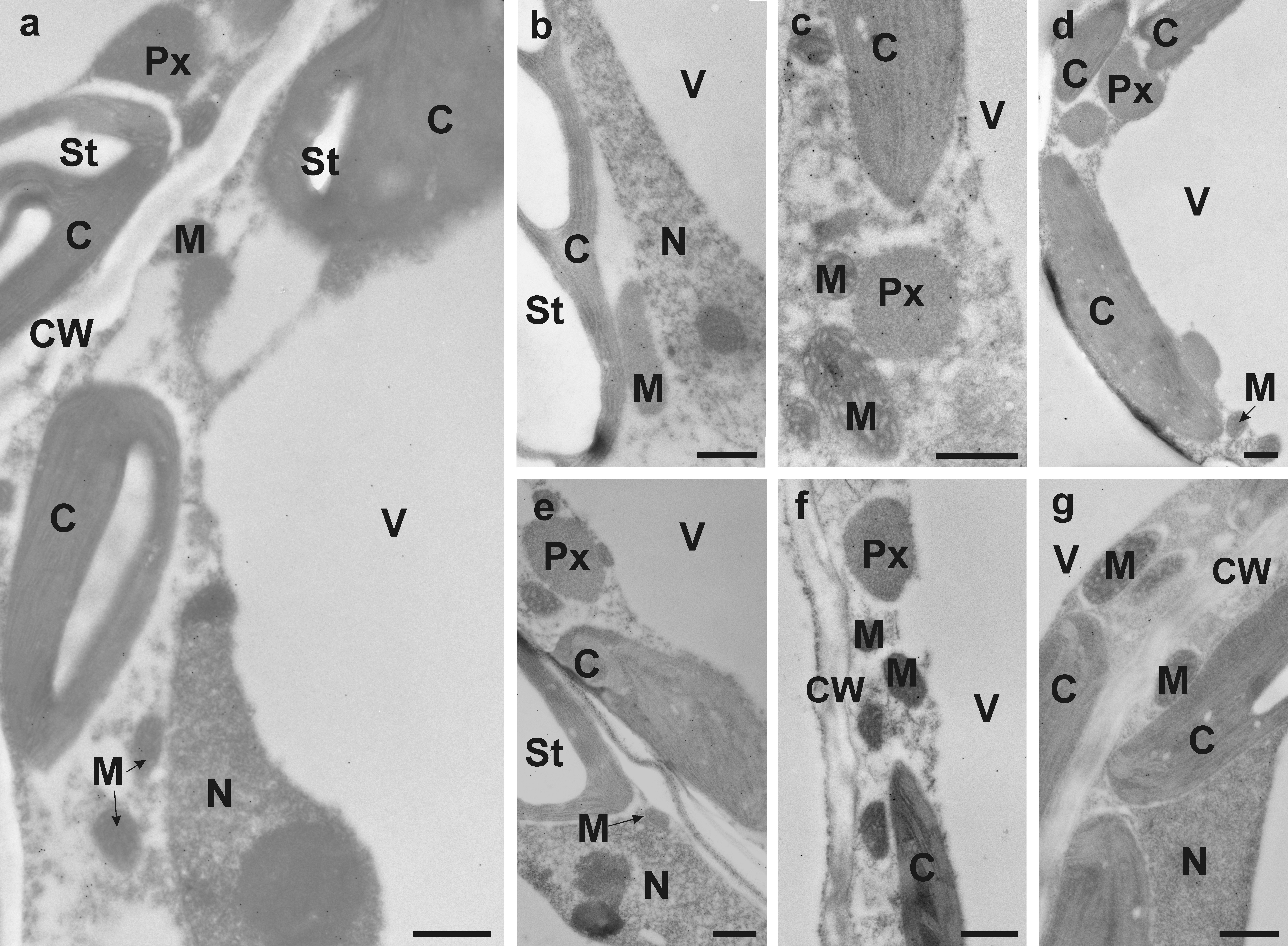

Supplement: Supplementary file 2 — Representative transmission electron micrographs showing gold particles bound to ascorbate on leaf sections from Arabidopsis thaliana pad2-1. Plants were treated with 0 (a), 50 (b–d), and 100 μM Cd (e–g) for 12 h (b, e), 96 h (c, f), and 14 days (a, d, g). Bars = 0.5 μm. C chloroplasts with or without starch (St), CW cell walls, M mitochondria, N nuclei, Px peroxisomes, V vacuoles (JPEG 5448 kb) [file 709_2013_576_MOESM2_ESM.jpg]

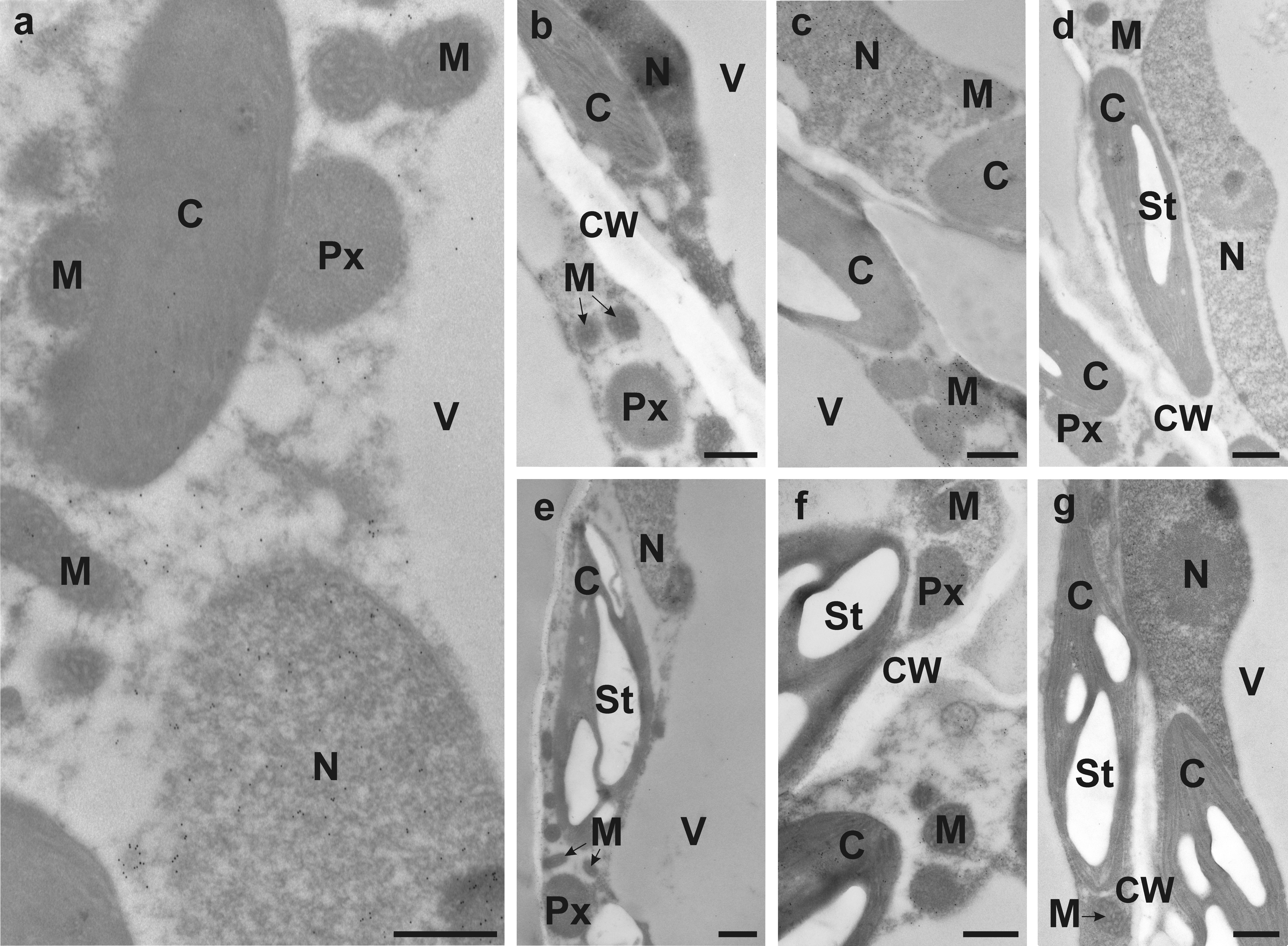

Supplement: Supplementary file 3 — Representative transmission electron micrographs showing gold particles bound to ascorbate on leaf sections from Arabidopsis thaliana vtc2-1. Plants were treated with 0 (a), 50 (b–d), and 100 μM Cd (e–g) for 12 h (b, e), 48 h (c, f), and 14 days (a, d, g). Bars = 0.5 μm. C chloroplasts with or without starch (St), CW cell walls, M mitochondria, N nuclei, Px peroxisomes, V vacuoles (JPEG 5514 kb) [file 709_2013_576_MOESM3_ESM.jpg]

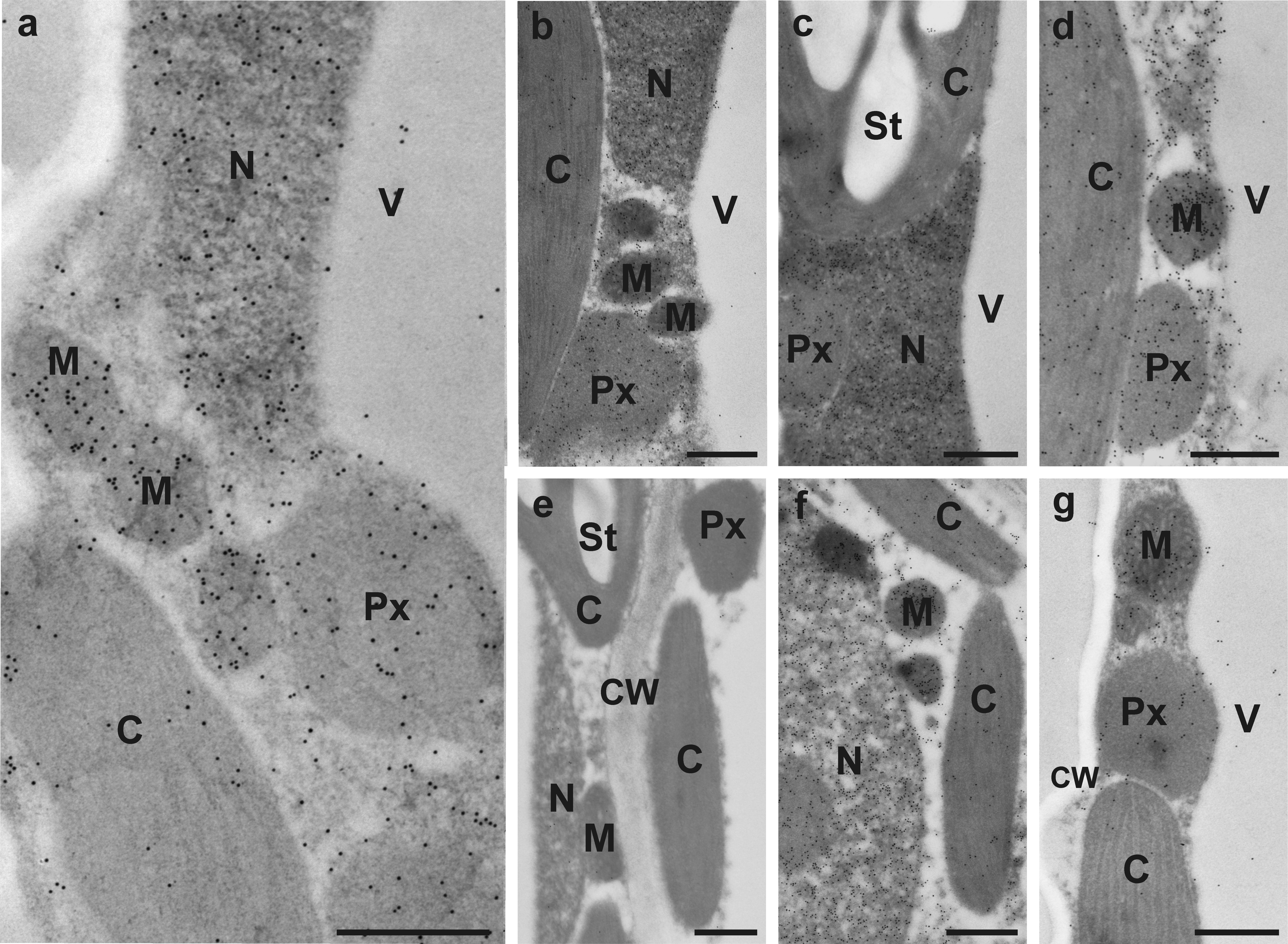

Supplement: Supplementary file 4 — Representative transmission electron micrographs showing gold particles bound to glutathione on leaf sections from Arabidopsis thaliana Col-0. Plants were treated with 0 (a), 50 (b–d), and 100 μM Cd (e–g) for 12 h (b, e), 48 h (c, f), and 14 days (a, d, g). Bars = 0.5 μm. C chloroplasts with or without starch (St), CW cell walls, M mitochondria, N nuclei, Px peroxisomes, V vacuoles (JPEG 5347 kb) [file 709_2013_576_MOESM4_ESM.jpg]

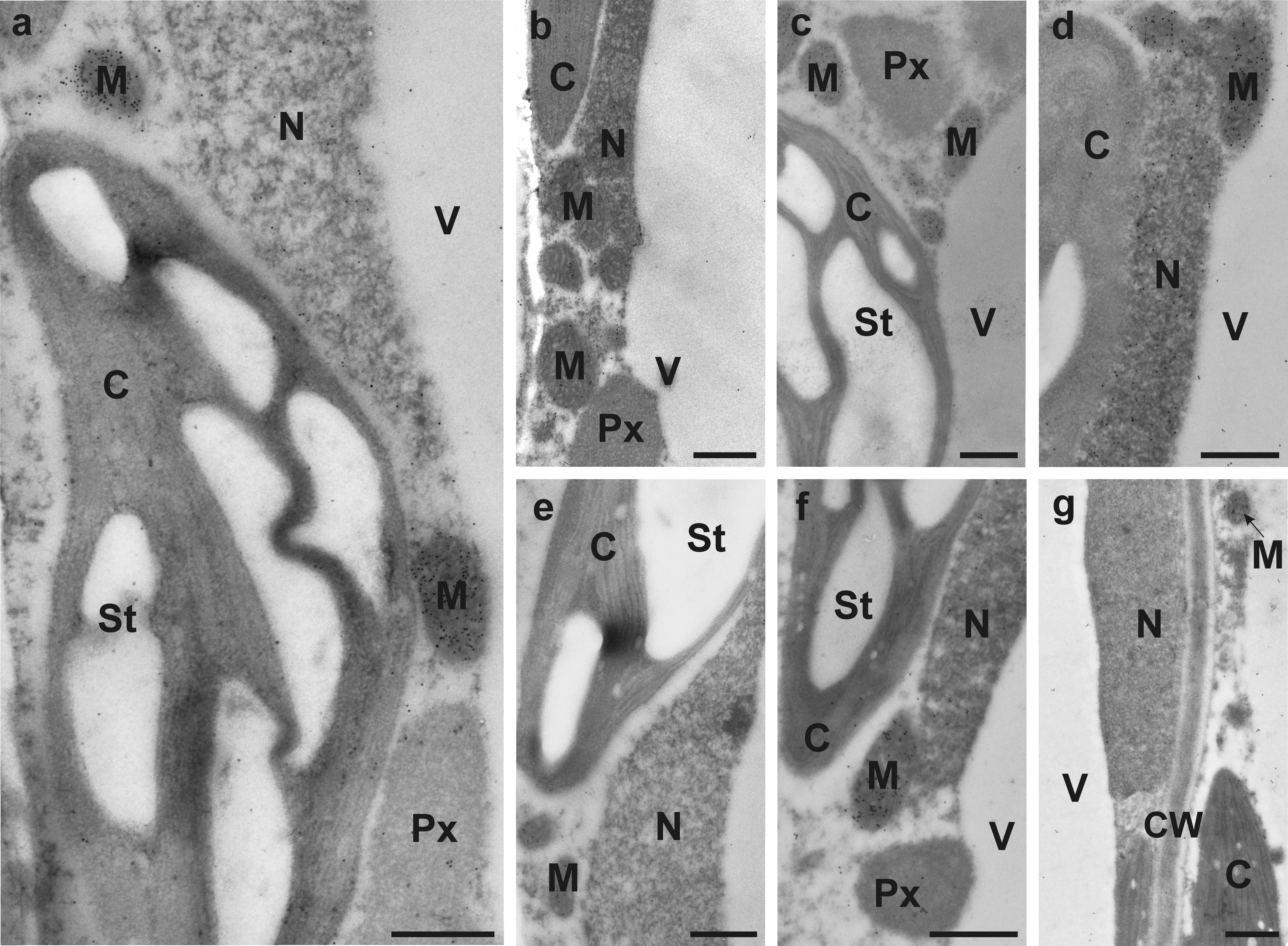

Supplement: Supplementary file 5 — Representative transmission electron micrographs showing gold particles bound to glutathione on leaf sections from Arabidopsis thaliana pad2-1. Plants were treated with 0 (a), 50 (b–d), and 100 μM Cd (e–g) for 12 h (b, e), 96 h (c, f), and 14 days (a, d, g). Bars = 0.5 μm. C chloroplasts with or without starch (St), CW cell walls, M mitochondria, N nuclei, Px peroxisomes, V vacuoles (JPEG 5448 kb) [file 709_2013_576_MOESM5_ESM.jpg]

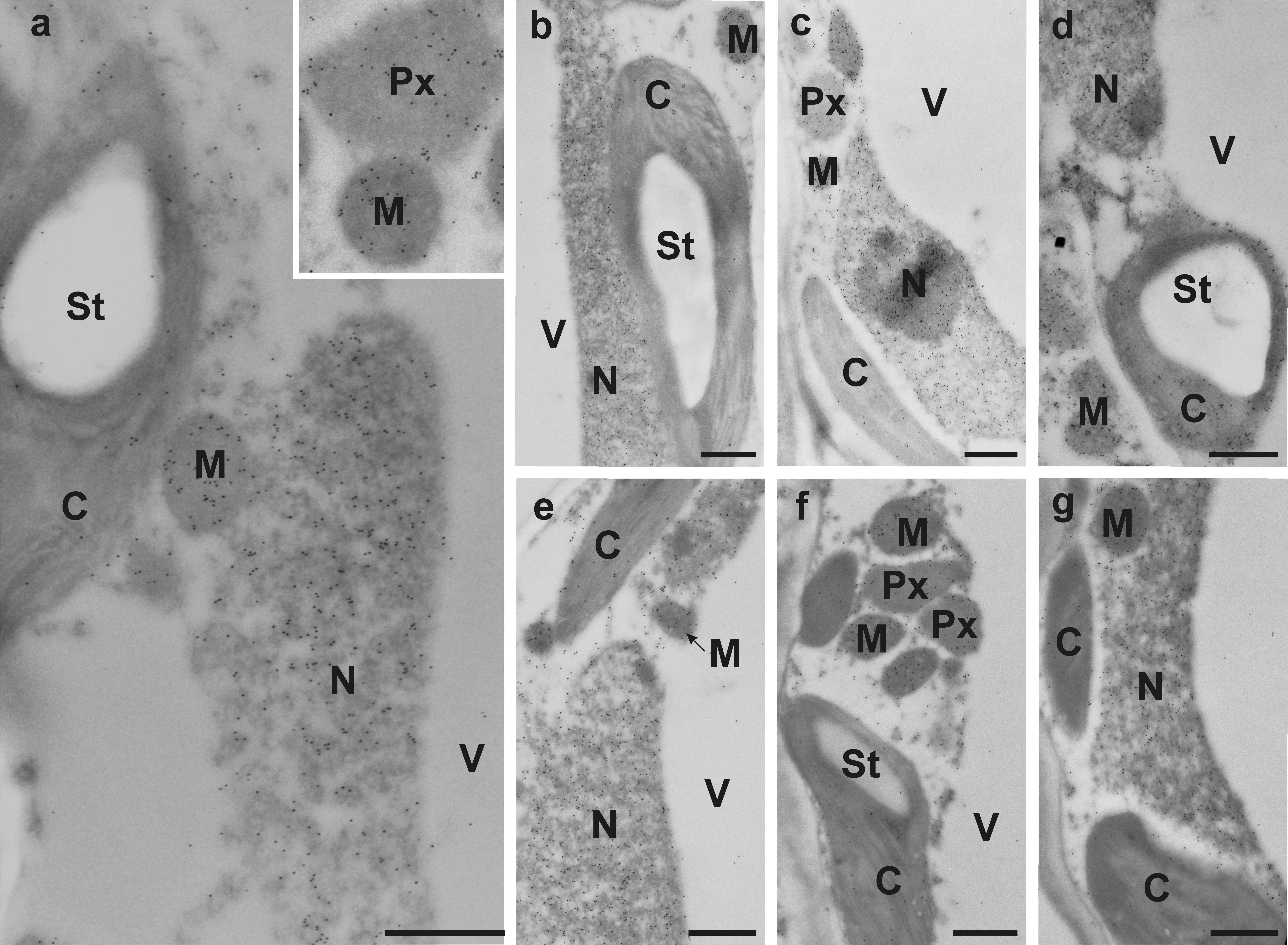

Supplement: Supplementary file 6 — Representative transmission electron micrographs showing gold particles bound to glutathione on leaf sections from Arabidopsis thaliana vtc2-1. Plants were treated with 0 (a), 50 (b–d), and 100 μM Cd (e–g) for 12 h (b, e), 48 h (c, f), and 14 days (a, d, g). Bars = 0.5 μm. C chloroplasts with or without starch (St), CW cell walls, M mitochondria, N nuclei, Px peroxisomes, V vacuoles (JPEG 5246 kb) [file 709_2013_576_MOESM6_ESM.jpg]
